# Supplementary material for: The composition and organization of Drosophila heterochromatin are heterogeneous and dynamic
Source: eLife. 2016 Aug 11;5:e16096. doi: 10.7554/eLife.16096 (PMC4981497; doi:10.7554/eLife.16096)
Supplement: Table 1—source data 3. — Silver-stained gel of a single step purification from S2 cells stably expressing FS-HP1a (lanes 1–3) or WT (lane 4) S2 cells. HP1a was purified in the absence of IR (lane 1), and 10 (lane 2) and 60 (lane 3) minutes after 10 Gy exposure. The HPips identified did not change significantly with respect to irradiation, therefore all purifications were used to identify candidate hits. DOI: http://dx.doi.org/10.7554/eLife.16096.007 [file elife-16096-table1-data3.pdf]

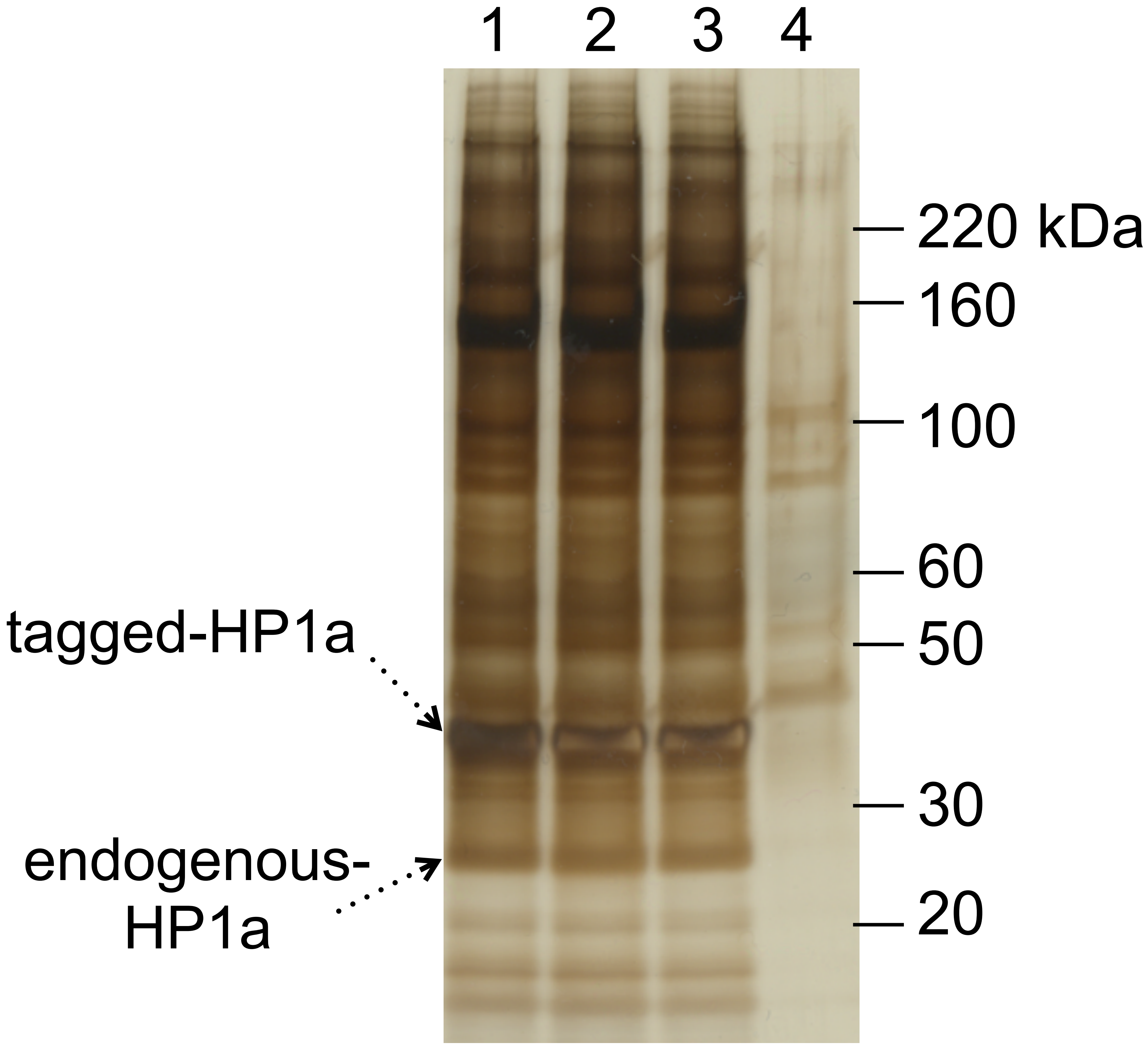

Table 1-source data 3: HP1a interacts with a large set of proteins
Silver-stained gel of a single step purification from S2 cells stably expressing FS-HP1a (lanes 1-3) or WT (lane 4) S2 cells. HP1a was purified in the absence of IR (lane 1), and 10 (lane 2) and 60 (lane 3) minutes after 10 Gy exposure. The HPips identified did not change significantly with respect to irradiation, therefore all purifications were used to identify candidate hits.
